# Comparison of ‘Mental training’ and physical practice in the mediation of a structured facial examination: a quasi randomized, blinded and controlled study

**DOI:** 10.1186/s12909-021-02603-0

**Published:** 2021-03-23

**Authors:** Arne Nelskamp, Benedikt Schnurr, Alexandra Germanyuk, Jasmina Sterz, Jonas Lorenz, Robert Sader, Miriam Rüsseler, Lukas B. Seifert

**Affiliations:** 1 Department of Oral, Cranio-Maxillofacial, and Facial Plastic Surgery, Goethe University, Theodor-Stern-Kai 7, 60590 Frankfurt, Germany; 2 Department of Urology, Medical Faculty of Saarland University, Saarbrücken, Germany; 3 Department of Trauma, Reconstructive and Hand Surgery, Goethe University, Frankfurt, Germany

## Abstract

**Background:**

The correct performance of a structured facial examination presents a fundamental clinical skill to detect facial pathologies. However, many students are not adequately prepared in this basic clinical skill. Many argue that the traditional ‘See One, Do One’ approach is not sufficient to fully master a clinical skill. ‘Mental Training’ has successfully been used to train psychomotor and technical skills in sports and other surgical fields, but its use in Oral and Maxillofacial Surgery is not described. We conducted a quasi-experimental to determine if ‘Mental Training’ was effective in teaching a structured facial examination.

**Methods:**

Sixty-seven students were randomly assigned to a ‘Mental Training’ and ‘See One, Do One’ group. Both groups received standardized video instruction on how to perform a structured facial examination. The ‘See One, Do One’ group then received 60 min of guided physical practice while the ‘Mental Training’ group actively developed a detailed, stepwise sequence of the performance of a structured facial examination and visualized this sequence subvocally before practicing the skill. Student performance was measured shortly after (T1) and five to 10 weeks (T2) after the training by two blinded examiners (E1 and E2) using a validated checklist.

**Results:**

Groups did not differ in gender, age or in experience. The ‘Mental Training’ group averaged significantly more points in T1 (pE1 = 0.00012; pE2 = 0.004; dE1 = 0.86; dE2 = 0.66) and T2 (pE1 = 0.04; pE2 = 0.008, dE1 = 0.37; dE2 = 0.64) than the ‘See One, Do One’ group. The intragroup comparison showed a significant (pE1 = 0.0002; pE2 = 0.06, dE1 = 1.07; dE2 = 0.50) increase in clinical examination skills in the ‘See One, Do One’ group, while the ‘Mental Training’ group maintained an already high level of clinical examination skills between T1 and T2.

**Discussion:**

‘Mental Training’ is an efficient tool to teach and maintain basic clinical skills. In this study ‘Mental Training’ was shown to be superior to the commonly used ‘See One, Do One’ approach in learning how to perform a structured facial examination and should therefore be considered more often to teach physical examination skills.

**Supplementary Information:**

The online version contains supplementary material available at 10.1186/s12909-021-02603-0.

## Background

Practical skills must be learned by every medical student regardless of her or his future specialization [1]. However, studies show that students consider the training of practical skills in the context of their medical education as insufficient [2] and there are still shortcomings in the training of basic clinical skills such as the physical examination in undergraduate medical education [3–5]. Although, many authors have demonstrated deficits in the training of clinical skills, the methodological approaches in teaching methods to impart competencies in those skills are not completely elaborate. Therefore, it is necessary to find effective educational strategies for each clinical skill.

In medical education, clinical skills are traditionally taught using the ‘See One, Do One’ method. In 1889, Halsted introduced a system in which medical students completed a university-sponsored, hospital-based surgical training program [6]. Halsted’s model of ‘See One, Do One, Teach One’ is based on acquiring increasing amounts of responsibility that culminate in near independence. Halsted was not only interested in developing a system to train surgeons, but also in creating teachers and role models [7]. Today this approach is labelled as the main component of clinical-bedside teaching. Students learn by watching an expert explaining and demonstrating a skill. This is followed by the first independent performance of the skill, which is mostly with a patient [7]. Although this method has been obviously proven to be effective, reality is forcing us to establish new teaching methods. The enormous workload including reduced lengths of stay in hospital combined with increasing case numbers, growing multimorbidity and increased complexity of treatments leads to significantly reduced chances for bedside teaching [8]. Often, the important last step ‘Teach one’ is often omitted or is undertaken without supervision in everyday clinical practice. Further, the health care system is facing increased economic pressure and scarcity of resources [9].

Thus, it would be optimal to establish a teaching method that does not involve many resources. Recently there has been a growing understanding that cognitive abilities, such as problem solving and movement-planning, play a crucial role in learning practical skills [10, 11]. This has resulted in a shift away from training methods that exclusively focus on the acquisition of motor skills but rather target the actual thought process when performing a clinical skill. This way of cognitive or mentally training in a certain skill has long been established in sports [12, 13] and has also been shown to be beneficial in the retrieval of motor abilities in rehabilitative medicine [14]. A reason discussed for its effectiveness resides in Janerod’s simulation theory [15] which hypotheses that the motor system is also part of a bigger cognitive network which includes various psychological activities. During this ‘Mental Training’, similar neural pathways are activated and similar changes in the brain take place as when actually performing a motor skill as functional MRI investigations have shown [15, 16]. This explains why ‘Mental Training’ can directly improve motor skills and induce a similar neuroplasticity as physical practice and thus can help to close the gap between the observation and the execution of a skill [17].

‘Mental Training’ has already been used successfully in the mediation of clinical skills and surgical training [17–20]. An influential study by Arora et al. [21] showed that participants who practiced with a standardized mental imaginary script showed greater improvements in learning laparoscopic cholecystectomies compared to a control group that participated in online lecture training. These results were supported by Immenroth et al., who demonstrated that mental rehearsal led to significantly better performance results compared to practical training in the mediation of laparoscopic cystectomies [19]. Despite these results, not all studies report a beneficial effect from ‘Mental Training’. Studies by Jungmann et al. and Sanders et al., for example, found no significant effect of ‘Mental Training’ compared to physical practice in the acquisition of laparoscopic knot-tying and basic surgical skills [22, 23].

In 2015, Rao et al. published a meta-analysis investigating ‘Mental Training’ in medical under- and postgraduate education for the training of practical skills [24]. Their analysis showed a great heterogeneity regarding the chosen methodology and design of the training process and also the skills that had to be learned widely differed. Furthermore, it is unclear if ‘Mental Training’ really presents an advantage in the training of clinical skills compared to traditional teaching methods.

Therefore, the aim of this study was to prospectively investigate the teaching efficacy of two teaching methods, namely a ‘Mental Training’ approach (study group) and the traditional ‘See One, Do One’ approach (control group) in the short- and long-term acquisition of a basic clinical skill in the field of Oral- and Maxillofacial Surgery, namely the structured facial examination of the head and face. Another aim of this study was to investigate the curricular (‘in vivo’) feasibility of the ‘Mental Training’ approach. The hypothesis of this study was that ‘Mental Training’ would be equal compared to the ‘See One, Do One’ approach.

## Methods

### Study design and participants

**Fig. 1:**
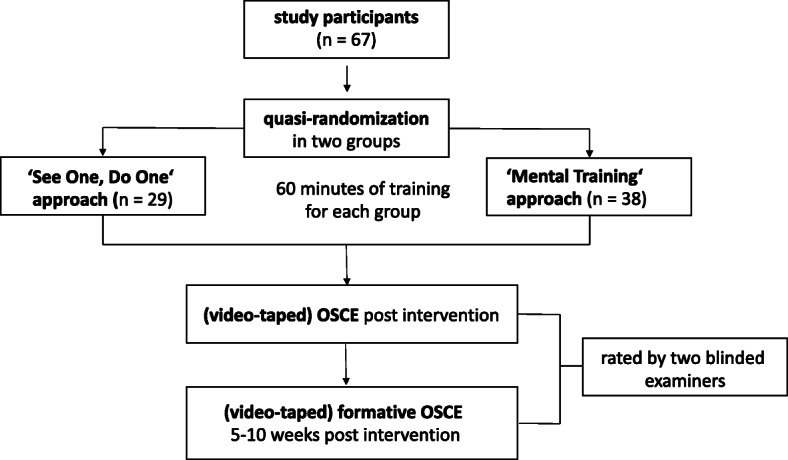
Study design and execution

Overall, 67 (female *n* = 49; male *n* = 18) 4th year dentistry students on a five-year program without previous experience in the field of Oral and Maxillofacial Surgery were assigned quasi-randomly to a ‘Mental Training’ group and a ‘See One, Do One’ group which was regarded as a control (Fig. 1). Participation in the study was voluntary and took place after written informed consent, which was revocable at any time. Students were blinded in relation to their knowledge of the didactic principles used during their training as well as affiliation to any study group. Basic data regarding student age, sex, and duration of study were collected using a questionnaire.

The study was conducted according to the ethical principles of the Helsinki Declaration (Ethical Principles for Medical Research Involving Human Subjects), and the local ethics committee noted that no further approval was necessary.

### Assignment of the students to the instructional approaches

The assignment of students to one of the learning groups with a maximum of 6 students per week occurred prior to the Oral and Maxillofacial Surgery apprenticeship independent of the authors and independent of study participation by the dean’s office. Groups were then assigned alternately within the 10-week span of the apprenticeship to one of the instructional approaches. This quasi-randomized allocation of students was chosen due to the curricular ‘in-vivo’ study design and intended to produce similar groups.

### Study protocol

The study was carried out within the Oral and Maxillofacial Surgery apprenticeship for dentistry students, which includes a five-day rotation through every section of the Department of Oral, Cranio-Maxillofacial and Facial Plastic Surgery, i.e. the operative room, the outpatient clinic or the emergency department. Before starting their rotation, students have to complete a *practical skills training* [25]*.* The aim is to give dentistry students a short overview of the most common reasons for consultation in Oral and Maxillofacial Surgery and prepare them for the upcoming clinic rotation. It is divided into a theoretical part (240 min) in the morning and practical skills training (240 min), during which the study took place, in the afternoon. Trained practical skills include performing a structured facial examination, placing a venous catheter in the context of an emergency treatment and placing an ‘Ernst ligature’ on a phantom model. Lessons were held in small groups ranging from five to six students.

### ‘See One, Do One’ approach

As quality assurance and standardization for the demonstration of a structured facial examination, the skill was videotaped based on the existing manual and checklist. The trainer demonstrated the video and explained the performance of a structured facial examination step-by step in detail. The explanations were predetermined in the manual and trained in the tutor training, which was identical for both approaches. Subsequently, students practiced the skill on each other under supervision and, if needed, correction of the tutor. Each student was advised to perform the structured facial examination at least once. In total the training lasted for 60 min.

### ‘Mental training’ approach

For the ‘Mental Training’ approach, the students received the same standardized video instruction of a structured facial examination. Subsequently, students practiced the skill under the supervision and, if needed, correction of the tutor for 30 min. Students then actively developed an individual detailed, stepwise sequence of the performance of a structured facial examination under the supervision and review of the tutor. Students were free to divide the skill into individual sub-steps which they noted on index cards. This process of creating an individual ‘mental map’ was supervised by tutor who would integrate missing steps or correct the sequence, if necessary. Students then visualized and internalized every step of the individual mental map in subvocal for 30 min. In total the training lasted for 60 min.

### Performance measurement

In order to assess the acquired competence in performing a structured facial examination, the OSCE-format was used during the training week directly after the intervention in the last 60 min of the practical skills training (T1) as a single station OSCE and 5 to 10 weeks later (T2) as part of a curricular and formative Oral and Maxillofacial Surgery OSCE (8 stations in total). A three point scale scale was used (0 points for not done, 1 point for done, but incorrect, 2 points for done and correct) for the checklist, which was based on the checklist used in the tutor manual (Supplement 1). In total, the checklist consisted of 24 items, which equals a maximum score of 48 points. The checklists implemented had been primarily piloted in previous undergraduate trainings that were not related to the mental training approach. In addition, the content validity was ensured through its creation as part of an expert workshop with didactic and surgical experts as well as through repeated application and adaption in the context of previous studies [25, 26] and OSCE exams. A timeframe of 5 min to complete each OSCE station was given.

Both at T1 and T2, students were video-recorded (Camera System: Panasonic HC-X929) for later performance measurement by two independent, blinded examiners with different levels of experience (Rater 1: Oral and Maxillofacial Surgery resident in year four of a five-year training; Rater 2: Oral and Maxillofacial Surgery resident in year three of a five-year training).

#### Data analysis

Microsoft Office 2016 (Microsoft Office 2007,© Microsoft Corporation, Redmond, USA) for Mac and SPSS Statistics version 19 (IBM, Armonk, USA) were used for the statistical analysis and graphical display of data.

To test for a normal distribution of the data, the Shapiro-Wilks-Test was used. Since the test results for both groups were not normally distributed at all times, the Mann-*Whitney-U-Test for non-parametric data* was used to test for significant differences in learning success in the inter- and intragroup comparison at T1 and T2. Furthermore, effect sizes were calculated for T1 to T2 using Cohens d. Cohen’s d is defined as the difference between two means divided by a standard deviation for the data resulting in an unitless value that helps to interpret the effect size of observed results and hence the statistical power of a study. For most types of effect sizes, a larger absolute value indicates a stronger effect. Since the sample size (*n* = 67) of our study was relatively small, Cohen’s d was used as an additional control test since prior studies have shown significant test results alone are not sufficient to interpret data and draw conclusions from this data [27]. The inter-rater reliability was measured using Pearson’s correlation coefficient (r).

Since there was a gender imbalance between female and male study participants, a gender analysis was conducted to investigate the effect of this imbalance on the outcome of the different tests, comparing the results of all female and male study participants.

### Sample size estimation

Based on prior examination results from the years before the intervention, we estimated an average student performance of 70% with a standard deviation of 10% in the OSCE. Based on the following parameters (Mean ‘Mental Training’ = 33, Mean ‘See One, Do One’ = 30, SD = 5, alpha = 0.05, beta = 0.2) a sample size of 88 was calculated.

## Results

### Study participants

Overall, 67 (f = 49; m = 18) students agreed to participate in the study. A total of 29 students (m = 8, w = 21) belonged to the ‘See One, Do One’ group while 38 students (m = 11, w = 29) were trained using the ‘Mental Training’ approach. Students from the ‘See One, Do One’ group (average age = 25,2, average duration of study = 8,5 semesters) did not significantly differ in baseline characteristics compared to the ‘Mental Training’ group (average age = 24,8, average duration of study = 8,5 semesters). The gender distribution corresponded the gender distribution at Goethe University’s Dental School [27]. All students (f = 49; m = 18) participated in the curricular OSCE 5–10 weeks after the intervention. Both teaching interventions could be carried out in a curricular setting with a 100% participation rate in the given timeframe without any complications.

### Outcome measures

At T1, students who had been trained with the ‘Mental Training’ approach showed highly significant better results in the performance of a structured facial examination (Rater 1 *p* < 0.001; Cohen’s d = 0.86; Rater 2 *p* = 0.005; Cohen’s d = 0.66, r = 0.74) than students who had been trained with the ‘See One, Do One’ approach (Table 1). In the long-term retention test 5–10 weeks after the intervention (T2), students who had been trained with the ‘Mental Training’ approach again showed significantly better results than students who had been trained with the ‘See One, Do One’ approach (Rater 1 *p* < 0.05; Cohen’s d = 0.38; Rater 2 *p* = 0.008; Cohen’s d = 0.64, r = 0.64) (Table 1). The intragroup comparison between T1 and T2 revealed a significant increase in clinical examination skills in the ‘See One, Do One’ group (Rater 1 *p* < 0.001; Cohen’s d = 1.07; Rater 2 *p* = 0.06; Cohen’s d = 0.50) (Table 1). The ‘Mental Training’ group did not show a significant growth in clinical examination skills from T1 to T2 (Rater 1 *p* < 0.10; Cohen’s d = 0.35; Rater 2 p = 0.06; Cohen’s d = 0.63) (Table 1). However, this group was able to maintain a very high level of competence from T1 to T2 (Table 1).

**Table 1:** Average scores obtained by the ‘Mental Training’ (MT) and ‘See One, Do One’ (SODO) group in the OSCE (48 possible points) and corresponding significance levels. Data are presented as Mean ± SD

	Group	T1	T2	***P***-Value	Effect Size (Cohen’s d)
**Rater 1**	**MT**	38.9 (± 6.5)	40.1 (± 4.4)	0.10 (M)	0.35
	**SODO**	33.5 (± 5.9)	39.2 (± 4.6)	**0.0002** (M)	1.07
	**P-Value**	**0.00012** (M)	**0.04** (M)		
	**Effect Size (Cohen’s d)**	0.86	0.38		
**Rater 2**	**MT**	37.7 (± 6.0)	40 (± 5.9)	0.06 (M)	0.63
	**SODO**	33.6 (± 6.5)	36.6 (± 5.5)	0.06 (M)	0.50
	**P-Value**	**0.004** (M)	**0.008** (M)		
	**Effect Size (Cohen’s d)**	0.66	0.64		

(M) = Mann-Whitney-White U test for ordinally distributed data

(T1) = directly after the intervention, (T2) = five to ten weeks after the intervention

### Gender analysis

Overall, no significant differences were found between female and male students at T1 and T2. Only Rater 1 found a significant difference at T1 between both genders (Table 2).

**Table 2:** Average scores obtained by female and male students in the OSCE (48 possible points) and corresponding significance levels. Data are presented as Mean ± SD

	Gender	T1	T2	P-Value	Effect Size (Cohen’s d)
**Rater 1**	**male**	35.3 (± 5.6)	38.1 (± 4.2)	**0.0005** (M)	0.56
	**female**	36.4 (± 6.5)	40.6 (± 4.6)	**0.002** (M)	0.74
	**P-Value**	**0.03** (M)	0.06 (M)		
	**Effect Size (Cohen’s d)**	0.18	0.56		
	**Effect Size (Cohen’s d)**	0.18	0.56		
**Rater 2**	**male**	35.8 (± 4.9)	37.3 (± 6.4)	**0.05** (M)	0.26
	**female**	35.5 (± 6.6)	38.7 (± 5.5)	**0.02** (M)	0.52
	**P-Value**	0.11 (M)	0.13 (M)		
	**Effect Size (Cohen’s d)**	0.05	0.23		

(M) = Mann-Whitney-White U test for ordinally distributed data

(T1) = directly after the intervention, (T2) = five to ten weeks after the intervention

## Discussion

The correct performance of a structured facial examination represents a fundamental basic clinical skill that is of great importance for the ongoing physician because of the high frequency of craniofacial trauma (48.1% of all injuries with an Abbreviated Injury Scale > 3) [28, 29] especially since previous studies have shown significant shortcomings regarding Oral and Maxillofacial Surgery-related knowledge and skills in undergraduate education [30–32]. The aim of this study was to prospectively investigate the teaching efficacy of two teaching methods, namely a ‘Mental Training’ approach (study group) and the traditional ‘See One, Do One’ approach (control group) in the short- and long-term acquisition of the above-mentioned basic clinical skill. Another aim of this study was to investigate the curricular (‘in vivo’) feasibility of the ‘Mental Training’ approach.

Overall, our results revealed significant performance differences between both groups in the short-term examination (T1) in favor of the ‘Mental Training’ group. Furthermore, the examination 5–10 weeks later (T2) revealed a significantly better long-term learning retention of the acquired practical skills for this group. Students in the control group, however, had also significantly improved their level of competence by the long-term comparison. The implementation of the ‘Mental Training’ approach in a curricular setting was completely feasible within the given timeframe for the Oral and Maxillofacial Surgery apprenticeship for dentistry students.

Interestingly, the control group had significantly improved its performance by the long-term examination at T2 while the study group managed to maintain a high performance level. The performance assessment at T2 as part of a curricular and formative surgical OSCE might have led to led to the significant performance improvement of the control-group in the long-term comparison measured. Raupach et al. described this phenomenon before and found that an examination itself can significantly enhance student performance independent of the chosen instructional approach [33].

We believe a possible reason for the found performance differences in favor of the ‘Mental Training’ approach in the short- and long-term measurement is the active visualization and verbalization in subvocal of the previously defined steps for a structured facial examination. The active imagination of the performance of a complex motor skill might be a reason for the better performance by the study group since students were forced to cognitively process and memorize each step of the skill before performing it. Previous studies came to similar conclusions [15–17]. For example, Janerod et al. hypothesized in their simulation theory that the motor system is part of a bigger cognitive network which includes various psychological activities [15]. They concluded that during mental training for a motor skill, similar neural pathway are activated and similar changes in the brain take place as when actually performing a motor skill. This hypothesis has been supported by functional MRI investigations. For example Seiler et al. could show similar brain activation patterns during arm rotation tasks while mentally training them [16]. This could explain why ‘Mental Training’ led to a significant improvement in our study group and was even shown to be superior compared to the control group regarding the acquisition of a basic clinical skill since it not only induced a similar neuroplasticity as physical practice in the ‘See One, Do One’ approach but may also have helped to close the gap between the observation and the execution of a complex skill [17].

There is a great heterogeneity regarding the impact of ‘Mental Training’ in surgical education in the current literature. There are randomized and controlled studies that have shown that Mental Training led to significantly better performance results in the mediation of laparoscopic cystectomies [19, 21]. However, not all studies that investigated ‘Mental Training’ in surgical education reported beneficial effects. In the mediation of laparoscopic knot-tying or surgical suturing and knot-tying no significant effect of ‘Mental Training’ compared to physical practice could be found [22, 23]. A possible explanation for this heterogeneity of outcome measures regarding ‘Mental Training’ might be the degree of complexity of a certain trained motor skill. Compared to the performance of a structured facial examination or the performance of a laparoscopic cystectomy, there are fewer steps when performing a simple surgical suture which also leaves less room for mistakes. This difference in complexity might explain why ‘Mental Training’ is especially suitable for more complex motor skills. Future studies have to investigate for what degree of complexity and which motor skills in particular mental training is superior compared to physical practice or other approaches like the use of teaching associates [34].

Overall, we could show that ‘Mental Training’ is significantly more effective than the traditional ‘See One, Do One’ approach in training for a structured facial examination and also led to significantly better knowledge retention in the long-term. Moreover, we demonstrated a ‘Mental Training’ protocol that was feasible in a curricular ‘in vivo’ setting with a given timeframe for teaching small groups.

### Limitations and strengths

Due to the limited curricular timeframe an objective assessment prior to the intervention could not be carried out which can be viewed as shortcoming of our study. However, both groups were naïve in terms of their knowledge on how to perform a structured facial examination and did not significantly differ in gender, age or academic success, which indicates that they were equivalent prior to the intervention. The sample size (*n* = 67 students) might be another limitation to the statistical power of the study.

However, compared to other studies, the present study was quasi-randomized, controlled, blinded, carried out within a curricular framework and assessed student performance over a six- to ten-week span. Moreover, it included an entire cross-section of an 8th semester at an accredited dental school. Future studies have to investigate whether our results can be transferred to other subjects and faculties.

## Conclusion

To our knowledge, this study is the first to have compared ‘Mental Training’ with the often used ‘See One, Do One’ approach in the mediation of a structured facial examination within a curricular framework.

‘Mental Training’ is significantly more effective than the ‘See One, Do one’ approach in the mediation of a structured facial examination in the short-and long-term and its implementation within a larger scale of students was completely possible.

## Supplementary Information

**Additional file 1: Supplement 1.** OSCE Checklist – Structured Facial Examination.

## Data Availability

The datasets used and/or analyzed during the current study are available from the corresponding author on reasonable request.

## Abbreviation

OSCE: Objective Structured Clinical Examination
